# A 5-year recurrence-free survivor with over ten colorectal liver metastases undergoing FOLFOX plus bevacizumab followed by two-stage hepatectomy

**DOI:** 10.1186/s40792-015-0113-6

**Published:** 2015-10-29

**Authors:** Yuka Tamaoki, Toru Beppu, Yasuo Sakamoto, Katsunori Imai, Hiromitsu Hayashi, Hidetoshi Nitta, Daisuke Hashimoto, Yuji Miyamoto, Yutaka Tsuruta, Akira Chikamoto, Hideo Baba

**Affiliations:** Department of Gastroenterological Surgery, Graduate School of Life Sciences, Kumamoto University, 1-1-1 Honjo, Chuo Ku, Kumamoto, 860-8556 Japan; Department of Multidisciplinary Treatment for Gastroenterological Cancer, Kumamoto University, 1-1-1 Honjo, Chuo Ku, Kumamoto, 860-8556 Japan

**Keywords:** A large number of colorectal liver metastases, Bevacizumab plus FOLFOX therapy, Two-stage hepatectomy

## Abstract

A 62-year-old male was admitted because of lower left abdominal pain and diarrhea. The patient was diagnosed with rectal cancer and multiple liver metastases. First, the laparoscopic Hartmann operation with a D3 lymph node dissection was performed. After five cycles of folinic acid, 5-fluorouracil and oxaliplatin (FOLFOX) and bevacizumab, and one additional FOLFOX, the tumor markers dramatically decreased; with carcinoembryonic antigen levels ranging from 1096.3 to 7.6 ng/ml and carbohydrate antigen 19–9 levels ranging from 3248.0 to 42.1 U/ml. Computed tomography showed a bilateral 14 colorectal liver metastases which indicated stable disease by the Response Evaluation Criteria In Solid Tumors (RECIST) criteria and optimal morphologic response. A two-stage hepatectomy was performed to complete a curative resection because of the insufficient remnant liver volume. Five partial hepatic resections in the left liver and the right portal vein ligation were performed during the first operation. Thirty-four days later, a right hepatectomy was successfully performed. Pathologically, there was tumor necrosis in 90 percent of the area of the metastasized liver, and viable cells were detected in only a marginal part of the liver. The patient had an uneventful postoperative course and was discharged fifteen days after the second operation. Uracil-tegafur plus leucovorin was administered for 6 months as an adjuvant chemotherapy treatment. The patient is currently alive and has remained disease-free for more than 5 years. In conclusion, an ideal combination of perioperative chemotherapy and curative resection may provide a chance of long-term survival without recurrence of disease for selected patients with more than ten bilateral colorectal liver metastases.

## Background

A large number of liver metastases have the greatest impact on disease-free survival (DFS) and overall survival (OS) for patients with colorectal liver metastases (CRLM) who underwent upfront hepatic resection [1]. Tumor number separation in patients with CRLM after hepatic resection should be accomplished using the 1–4 and ≥5 classification [2]. Eight or more CRLM values have shown significantly worse recurrence-free survival (RFS) rates than CRLM values that are between 1 and 7 (28.7 % versus 13.6 %), partly due to a greater rate of hepatic recurrence (57.7 % versus 37.1 %) [3]. In 102 CRLM patients with 8 or more tumors who underwent upfront hepatectomy without chemotherapy, the 5-year OS rate of 33 % was relatively good, but the 5-year RFS rate was only 1.7 % [4].

Two-stage hepatectomy has been developed as a surgical modality for patients with multiple and bilateral CRLM. Five-year RFS and median RFS values were only 11 % and 9.4 months, respectively, instead of 53 (87 %) out of the 61 patients attempting the two-stage hepatectomy and received the preoperative chemotherapy, and 20 (87 %) out of the 23 patients who received the interval chemotherapy and completed the two-stage hepatectomy [5]. Induction chemotherapy renders curative resection for unresectable or marginally resectable patients with CRLM [6, 7]; however, almost all patients with a large number of CRLM live with recurrence. Herein, we report a CRLM patient with 15 metastases who was treated with effective induction chemotherapy and precise hepatic resection. He has remained alive and without any recurrence of disease for more than 5 years.

## Case presentation

**Fig. 1:**
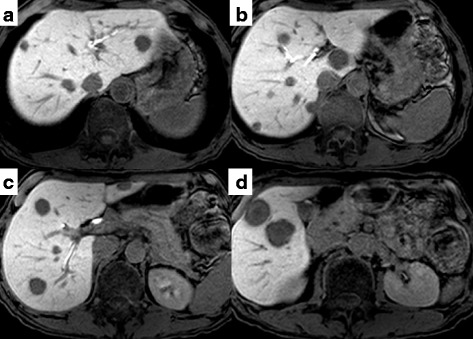
Gd-EOB-DTPA-enhanced MRI on hepato-biliary-phase findings of CRLM upon admission. **a**–**c** A large number of bilateral CRLM were detected on hepato-biliary-phase images of EOB-MRI as homogenous hypointensity. **d** The largest metastasis measured 2.5 cm in diameter on segment 5

**Fig. 2:**
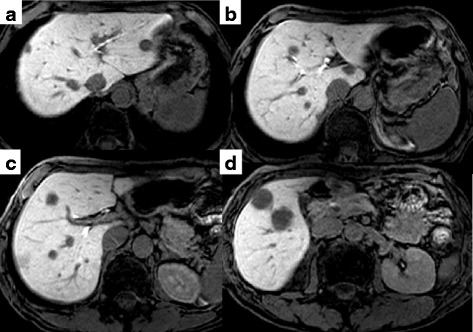
Gd-EOB-DTPA-enhanced MRI on hepato-biliary-phase findings of CRLM after the induction chemotherapy. **a–d** The volume of bilateral CRLM slightly downsized and the tumor border turned to be clear on hepato-biliary-phase images of EOB-MRI

**Fig. 3:**
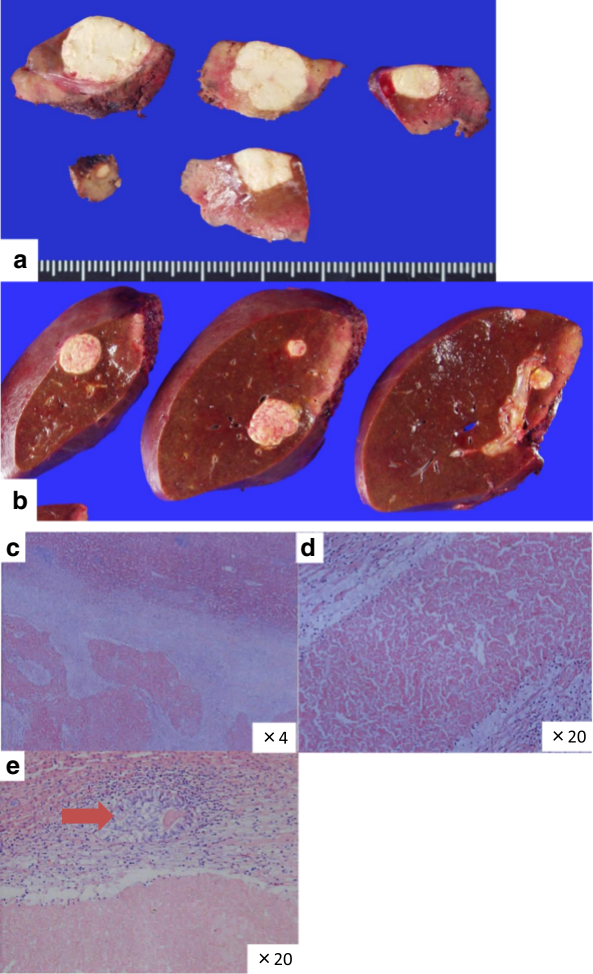
Macroscopic and histological findings of the resected specimen. The fresh resected specimen on the 1st stage (**a**) and 2nd stage (**b**) hepatectomy showed several whitish tumors with a sufficient surgical margin behind the normal liver. Almost all parts of the bilateral CRLM showed complete necrosis (**c**, **d**) (H&E staining, objective; ×4, ×20); however, viable cells were detected only on marginal parts of the 2nd resected liver metastases (**e**). (H&E staining, objective; ×20)

A 62-year-old male was admitted because of lower left abdominal pain and diarrhea. The laboratory data on admission showed a carcinoembryonic antigen (CEA) level of 189.1 ng/ml and a carbohydrate antigen 19–9 (CA19-9) level of 723.0 U/ml (Table 1). Colonoscopy showed stenosis by a rectal tumor. A magnetic resonance imaging (MRI) showed colorectal cancer with swelling of the primary lymph nodes and 15 bilateral CRLM (Fig. 1). First, the laparoscopic Hartmann operation with a D3 lymph node dissection was performed for primary rectal cancer. Pathologic diagnosis was moderately differentiated tubular adenocarcinoma. The pathologic findings of upper rectal cancer showed a tumor perforating to the surface of the visceral peritoneum, three metastases in primary lymph nodes, and a highly vascular space invasion. Final stages were T4a, N1b, M1a, and Stage IV in the 7th revision of the AJCC/ UICC TNM staging system. We enrolled this patient into a clinical trial of the Kyushu study group of clinical Cancer (KSCC0802 - UMIN000001308), a phase II study of induction chemotherapy followed by hepatic resection for unresectable or marginally resectable CRLM patients [8]. Following 5 cycles of 5-fluorouracil and oxaliplatin (FOLFOX) with bevacizumab, and one cycle of FOLFOX, the CEA level decreased from 1096.3 to 7.6 ng/ml (normal range ≤3.4 ng/ml), and CA19-9 level decreased from 3248.0 to 42.1 U/ml (normal range ≤37.0 U/ml). After the induction chemotherapy, a MRI showed that the volume of CRLM slightly reduced and number of those decreased from 15 to 14 (Fig. 2). However, it did not show so called “early tumor shrinkage” that is associated with long-term outcome in KRAS wild-type group-treated chemotherapy plus cetuximab [9]. According to the Response Evaluation Criteria in Solid Tumors (RECIST) [10] and the morphologic response criteria [11], the patient was assessed as stable disease and had an optimal morphologic response, respectively. The preoperative indocyanine green retention rate at a 15 min (ICG R 15) value and the uptake ratio of the liver to the liver plus heart at 15 min (LHL 15) were 9.6 and 0.96, respectively. The right hepatic resection rate was estimated to be 67.6 %. A two-stage hepatectomy instead of a general hepatectomy was thus conducted to complete a curative resection because of the insufficient remnant liver volume. Five partial hepatic resections of the left liver metastases including left S1 tumor and the right portal vein ligation were performed during the first stage. In the 2nd preoperative assessment, percent remnant functional liver volume was increased from 32.4 to 43.9 % by right portal vein ligation [12]. Thirty-four days after the first operation, a right hepatectomy was successfully performed. The operation time and blood loss was 295 min and 20 ml and 301 min and 435 ml, respectively, in the first and second operation. A blood transfusion was never required. Although nine metastases were resected, one metastasis had vanished by chemotherapy. Pathologically, we observed tumor necrosis in most of the area of the metastasized bilateral liver. Viable cells were partially mixed in the 1st resected liver tissues and were only present marginally in the 2nd resected ones (Fig. 3). The background liver showed mild sinusoidal obstruction induced by oxaliplatin without steatosis or steatohepatitis. The patient had an uneventful postoperative course and was discharged 15 days after the resection. Uracil-tegafur plus leucovorin (UFT/LV) was given for 6 months as an adjuvant chemotherapy treatment. The patient is now alive more than 5 years and without the recurrence of disease.

**Table 1:** The laboratory data on admission

WBC	7.5	×10^3^/μl,	BUN	11.2	mg/dl
RBC	4.95	×10^6^/μl	Creatinine	0.63	mg/dl
Hemoglobin	16.3	g/dL	Amylase	78	U/l
Hematocrit	48.6	%	Cholinesterase	270	U/l
Platelet	259	×10^3^/μl	Na	141	mEq/l
PT activity	101	%	K	4.1	mEq/l
AST	21	U/l	Cl	106	mEq/l
ALT	12	U/l	CRP	0.38	mg/dl
ALP	428	U/l	Glucose	91	mg/dl
LDH	245	U/l	HbA1c	5.3	%
γ-GTP	20	U/l	CEA	189.1	ng/ml
Total bile acid	0.6	μmol/l	CA19-9	723.0	U/ml
Total protein	7.3	g/dl	HBs-Ag	(−)	
Albumin	4.3	g/dl	HCV-Ab	(−)	

*WBC* white blood cell, *RBC* red blood cell, *PT* prothrombin time, *AST* aspartate aminotransferases, *ALT* alanine aminotransferases, *ALP* alkali phosphatase, *LDH* lactate dehydrogenase, *γ-GTP* γ-glutamyltransferase, *BUN* blood urea nitrogen, *CRP* C-reactive protein, *CEA* carcinoembryonic antigen, *CA19-9* carbohydrate antigen 19–9, *HBs-Ag* hepatitis B surface antigen, *HCV-Ab* anti-hepatitis C antibody

## Discussion

This patient might have been initially resectable by two-stage hepatectomy on admission; however, we avoided upfront hepatectomy, which was decided to be oncologically unsuitable. A project study group of Japanese Society of Hepato-Biliary-Pancreatic Surgery (JHBPS) nomogram can make a preoperative prediction of the DFS of CRLM treated with hepatic resection [1]. Six preoperative factors were selected to create the nomogram for DFS: synchronous metastases, 3 points; primary lymph node positive, 3 points; number of tumors 2–4, 4 points and ≥5, 9 points; largest tumor diameter >5 cm, 2 points; extrahepatic metastases at hepatectomy, 4 points, and preoperative CA19-9 level >100 U/ml, 4 points. This patient was evaluated at 19 points out of a total of 25 points from the positive variables of synchronous metastases, positive primary lymph node, metastases ≥5, and a preoperative CA19-9 level of >100. The data were instantly calculated using the DFS simulation program as a supplementary item. The estimated 5-year DFS and median DFS for this patient were quite low: 2 and 5.7 months, respectively. Nevertheless, the patient was able to survive without the recurrence of disease for over 5 years.

We enrolled this patient into a phase II clinical trial (KSCC0802) of induction chemotherapy followed by a hepatic resection for unresectable or marginally resectable CRLM [8]. Fourteen bilateral and scattered tumors were detected even after 5 cycles of FOLFOX and bevacizumab, followed by one cycle of FOLFOX. The tumor response was determined as stable disease by the RECIST; however, tumor markers dramatically decreased before the first hepatic resection. We have already reported that the normalization of tumor marker levels after chemotherapy was an important factor to obtain long-term survival after induction chemotherapy and hepatectomy [13]. This patient showed a distinct reduction of tumor markers by induction chemotherapy; the CEA level decreased from 1096 to 7.6 ng/ml, and the CA19-9 level decreased from 3248 to 42.1 U/ml. During chemotherapy, the half-life of CEA in this patient was 15 days, which is 20 days shorter than normal and also an indication of good prognosis [14]. Moreover, this patient showed an optimal morphologic response, which has been positively correlated with excellent OS rates, especially in patients with chemotherapy and bevacizumab [11]. Bevacizumab in combination with oxaliplatin/fluoropyrimidines can provide the highest major pathological response rate [15], and the pathological response to induction chemotherapy has been shown to correlate with improved survival [16]. In fact, this patient showed a Grade 2 response, which is defined as either necrosis or a disappearance of the tumor in >2/3 of the entire tumor.

This case was performed by a conventional two-stage hepatectomy in 2009. With another operation procedure, the associating liver partition and portal vein ligation for staged hepatectomy (ALPPS) may be one of the techniques in this case; however, this technique requires enough attention because of having high morbidity and mortality instead of a short-term hypertrophy and high resectability rates [17]. In this case, a traditional two-stage hepatectomy was successfully completed within a 34-day interval. Fortunately, the tumor number did not increase between the two operations. We observed no requirement of a blood transfusion and no morbidity in both hepatic resections. A large amount of intraoperative bleeding, a blood transfusion, or a major complication can shorten the postoperative survival rates [18, 19]. Although only six courses of FOLFOX can increase the morbidity after a hepatic resection [20], the combinatorial use of bevacizumab may affect the decrease of sinusoidal obstruction [21]. Fifty-seven days after the second surgery, the administration of UFT/LV was started and was continued for 6 months. The most recently used adjuvant chemotherapy treatment with the oral administration of UFT/LV for 6 months resulted in significantly better RFS rates compared to the hepatic resection alone [22].

## Conclusions

Suitable perioperative chemotherapy and curative hepatic resection may provide long-term survival rates without the recurrence of disease for selected patients with a large number of bilateral CRLM.

## Consent

Written informed consent was obtained from the patient for publication of this Case Report and any accompanying images.

## Abbreviations

CA19-9: carbohydrate antigen 19–9
CEA: carcinoembryonic antigen
CRLM: colorectal liver metastases
DFS: disease-free survival
FOLFOX: folinic acid, 5-fluorouracil and oxaliplatin
ICG R 15: indocyanine green retention rate at 15 min
JHBPS: Japanese Society of Hepato-Biliary-Pancreatic Surgery
KSCC: Kyushu study group of clinical cancer
LHL 15: the liver to the liver plus heart at 15 min
MRI: magnetic resonance imaging
OS: overall survival
RECIST: Response Evaluation Criteria in Solid Tumors
RFS: recurrence-free survival
UFT/LV: uracil-tegafur plus leucovorin
UMIN: University Hospital Medical Information Network

## References

[1] Beppu T, Sakamoto Y, Hasegawa K, Honda G, Tanaka K, Kotera Y (2012). A nomogram predicting disease-free survival in patients with colorectal liver metastases treated with hepatic resection: multicenter data collection as a Project Study for Hepatic Surgery of the Japanese Society of Hepato-Biliary-Pancreatic Surgery. J Hepatobiliary Pancreat Sci.

[2] Beppu T, Sakamoto Y, Hasegawa K, Honda G, Tanaka K, Kotera Y (2014). Optimal cut-off value for the number of colorectal liver metastases: a project study for hepatic surgery of the Japanese Society of Hepato-Biliary-Pancreatic Surgery. J Hepatobiliary Pancreat Sci.

[3] Viganò L, Capussotti L, Majno P, Toso C, Ferrero A, De Rosa G (2015). Liver resection in patients with eight or more colorectal liver metastases. Br J Surg.

[4] Saiura A, Yamamoto J, Hasegawa K, Koga R, Sakamoto Y, Hata S (2012). Liver resection for multiple colorectal liver metastases with surgery up-front approach: bi-institutional analysis of 736 consecutive cases. World J Surg.

[5] Narita M, Oussoultzoglou E, Jaeck D, Fuchschuber P, Rosso E, Pessaux P (2011). Two-stage hepatectomy for multiple bilobar colorectal liver metastases. Br J Surg.

[6] Adam R, Delvart V, Pascal G, Valeanu A, Castaing D, Azoulay D (2004). Rescue surgery for unresectable colorectal liver metastases downstaged by chemotherapy: a model to predict long-term survival. Ann Surg.

[7] Beppu T, Miyamoto Y, Sakamoto Y, Imai K, Nitta H, Hayashi H (2014). Chemotherapy and targeted therapy for patients with initially unresectable colorectal liver metastases, focusing on conversion hepatectomy and long-term survival. Ann Surg Oncol.

[8] Beppu T, Emi Y, Tokunaga S, Oki E, Shirabe K, Ueno S (2014). Kyushu Study group of Clinical Cancer (KSCC). Liver resectability of advanced liver-limited colorectal liver metastases following mFOLFOX6 with bevacizumab (KSCC0802 Study). Anticancer Res.

[9] Piessevaux H, Buyse M, Schlichting M, Van Cutsem E, Bokemeyer C, Heeger S (2013). Use of early tumor shrinkage to predict long-term outcome in metastatic colorectal cancer treated with cetuximab. J Clin Oncol.

[10] Therasse P, Arbuck SG, Eisenhauer EA, Wanders J, Kaplan RS, Rubinstein L (2000). New guidelines to evaluate the response to treatment in solid tumors. European Organization for Research and Treatment of Cancer, National Cancer Institute of the United States, National Cancer Institute of Canada. J Natl Cancer Inst.

[11] Shindoh J, Loyer EM, Kopetz S, Boonsirikamchai P, Maru DM, Chun YS (2012). Optimal morphologic response to preoperative chemotherapy: an alternate outcome end point before resection of hepatic colorectal metastases. J Clin Oncol.

[12] Beppu T, Hayashi H, Okabe H, Masuda T, Mima K, Otao R (2011). Liver functional volumetry for portal vein embolization using a newly developed 99mTcgalactosyl human serum albumin scintigraphy SPECT-computed tomography fusion system. J Gastroenterol.

[13] Sakamoto Y, Miyamoto Y, Beppu T, Nitta H, Imai K, Hayashi H, et al. Post-chemotherapeutic CEA and CA19-9 Are Prognostic Factors in Patients with Colorectal Liver Metastases Treated with Hepatic Resection After Oxaliplatin-based Chemotherapy. Anticancer Res. (in press) 25862901

[14] Nagai Y, Beppu T, Sakamoto Y, Miyamoto Y, Hayashi H, Nitta H (2014). Carcinoembryonic antigen half-life is an early predictor of therapeutic effects in induction chemotherapy for liver metastases from colorectal cancer. Anticancer Res.

[15] Wicherts DA, de Haas RJ, Sebagh M, Saenz Corrales E, Gorden DL, Lévi F (2011). Bevacizumab was demonstrated to enhance pathological response compared with chemotherapy alone. Br J Surg.

[16] Blazer DG, Kishi Y, Maru DM, Kopetz S, Chun YS, Overman MJ (2008). Pathologic response to preoperative chemotherapy: a new outcome endpoint after resection of hepatic colorectal metastases. J Clin Oncol.

[17] Alvarez FA, Ardiles V, de Santibanes M, Pekolj J, de Santibanes E (2015). Associating liver partition and portal vein ligation for staged hepatectomy offers high oncological feasibility with adequate patient safety: a prospective study at a single center. Ann Surg.

[18] Chikamoto A, Beppu T, Masuda T, Otao R, Okabe H, Hayashi H (2012). Amount of operative blood loss affects the long-term outcome after liver resection for hepatocellular carcinoma. Hepatogastroenterology.

[19] Farid SG, Aldouri A, Morris-Stiff G, Khan AZ, Toogood GJ, Lodge JP (2010). Correlation between postoperative infective complications and long-term outcomes after hepatic resection for colorectal liver metastasis. Ann Surg.

[20] Nordlinger B, Sorbye H, Glimelius B, Poston GJ, Schlag PM, Rougier P (2008). Perioperative chemotherapy with FOLFOX4 and surgery versus surgery alone for resectable liver metastases from colorectal cancer (EORTC Intergroup trial 40983): a randomised controlled trial. Lancet.

[21] Klinger M, Eipeldauer S, Hacker S, Herberger B, Tamandl D, Dorfmeister M (2009). Bevacizumab protects against sinusoidal obstruction syndrome and does not increase response rate in neoadjuvant XELOX/FOLFOX therapy of colorectal cancer liver metastases. Eur J Surg Oncol.

[22] Saiura A, Yamamoto J, Hasegawa K, Oba M, Takayama T, Miyagawa S (2014). A combination of oral uracil-tegafur plus leucovorin (UFT + LV) is a safe regimen for adjuvant chemotherapy after hepatectomy in patients with colorectal cancer: safety report of the UFT/LV study. Drug Discov Ther.

